# The Response Regulator YycF Inhibits Expression of the Fatty Acid Biosynthesis Repressor FabT in *Streptococcus pneumoniae*

**DOI:** 10.3389/fmicb.2016.01326

**Published:** 2016-08-25

**Authors:** Maria L. Mohedano, Mónica Amblar, Alicia de la Fuente, Jerry M. Wells, Paloma López

**Affiliations:** ^1^Laboratorio de Biología Molecular de Bacterias Gram positivas, Departamento de Microbiología Molecular y Biología de las Infecciones, Centro de Investigaciones Biológicas, Consejo Superior de Investigaciones CientíficasMadrid, Spain; ^2^Unidad de Patología Molecular del Neumococo, Centro Nacional de Microbiología, Instituto de Salud Carlos IIIMajadahonda, Spain; ^3^Host–Microbe Interactomics, Animal Sciences Department, University of WageningenWageningen, Netherlands

**Keywords:** two component systems, *Streptococcus pneumoniae*, fatty acid biosynthesis, essential response regulator, transcriptional regulation

## Abstract

The YycFG (also known as WalRK, VicRK, MicAB, or TCS02) two-component system (TCS) is highly conserved among Gram-positive bacteria with a low G+C content. In *Streptococcus pneumoniae* the YycF response regulator has been reported to be essential due to its control of *pcsB* gene expression. Previously we showed that overexpression of *yycF* in *S. pneumoniae* TIGR4 altered the transcription of genes involved in cell wall metabolism and fatty acid biosynthesis, giving rise to anomalous cell division and increased chain length of membrane fatty acids. Here, we have overexpressed the *yycFG* system in TIGR4 wild-type strain and *yycF* in a TIGR4 mutant depleted of YycG, and analyzed their effects on expression of proteins involved in fatty acid biosynthesis during activation of the TCS. We demonstrate that transcription of the *fab* genes and levels of their products were only altered in the YycF overexpressing strain, indicating that the unphosphorylated form of YycF is involved in the regulation of fatty acid biosynthesis. In addition, DNA-binding assays and *in vitro* transcription experiments with purified YycF and the promoter region of the *FabTH*-*acp* operon support a direct inhibition of transcription of the FabT repressor by YycF, thus confirming the role of the unphosphorylated form in transcriptional regulation.

## Introduction

Two-component signal transduction systems are wide spread in bacteria and participate in cell regulatory processes such as virulence, quorum sensing, chemotaxis, nutrient utilization, and cell cycle regulation, as well as adaptation to environmental changes. Typical two-component systems (TCSs) consist of two proteins, a sensor histidine kinase (HK) and a response regulator (RR) and are considered potential antibacterial drug targets (reviewed by Casino et al., 2010; Raghavan and Groisman, 2010; Gross and Beier, 2012; Velikova et al., 2013). In response to an appropriate stimulus, the HK or phosphatase activity of the HK is modulated, thereby transphosphorylating its cognate RR at its aspartyl residue and activating its binding to DNA operator sequences in specific promoters. In addition, the phosphorylation levels of the RR may be controlled by other specific aspartyl phosphatases (Silversmith, 2010; Huynh and Stewart, 2011). The RR typically contains two domains: a DNA-binding or effector domain, and a receiver domain that acts as a regulator of the effector domain. Activation of the RR protein results in a downstream effect, which may involve protein–protein interactions and/or affect gene regulation at the transcription level. Variation in the sequence of HK sensing domains and RR effector domains allows the conserved His–Asp phosphotransfer system to couple diverse input stimuli, such as nutrients, redox state, osmolarity, or antibiotics, to an equally diverse range of output responses (Mascher et al., 2006; Bem et al., 2015).

The YycG/YycF system also designated as WalK/WalR (Dubrac et al., 2008) is the most highly conserved TCS in gram-positive bacteria with low G+C content. YycF belongs to the OmpR/PhoB subfamily of response regulators, which are characterized by a winged helix–turn–helix effector domain involved in DNA binding. The OmpR/PhoB members possess similar structures for their receiver and effector domains, and also a common active dimer state that is believed to be conserved throughout the subfamily. Moreover, phosphorylation promotes the formation of dimers of many RRs including PhoB (review in Gao and Stock, 2010). The amino acid sequence of the region involved in dimerization (with α4-β5-α5 topology) is highly conserved within the OmpR/PhoB subfamily, including the pneumococcal YycF, whose receiver domain has been crystallized as a dimer (Bent et al., 2004).

The YycG/YycF systems of *Bacillus subtilis* (Fabret and Hoch, 1998), and of many Gram-positive pathogens including *Streptococcus pneumoniae* (TCS also designated as MicAB, VicRK and 492hkrr; Martin et al., 1999; Throup et al., 2000; Echenique and Trombe, 2001; Wagner et al., 2002; Dubrac and Msadek, 2004; Senadheera et al., 2005) have been investigated. In *B. subtilis* and *S. aureus* both YycF and YycG are essential (Fabret and Hoch, 1998; Martin et al., 1999), whereas in streptococci only the RR is essential (Lange et al., 1999; Throup et al., 2000; Senadheera et al., 2005). Unsuccessful attempts to inactivate the TCS in *Listeria monocytogenes, Enterococcus faecalis* and *Staphylococcus epidermidis* suggest that YycFG is also essential in these bacteria (Kallipolitis and Ingmer, 2001; Hancock and Perego, 2004; Qin et al., 2006). In addition, phosphorylation of the RR on aspartate residue 52 is required for pneumococcal growth (Ng et al., 2003). This essentiality in bacterial pathogens, coupled with the fact that TCSs have not been found in humans, makes the YycFG system an attractive target for developing antimicrobial compounds (Dubrac et al., 2008; Velikova et al., 2013; Bem et al., 2015). In fact, walrycin A and walrycin B, which inhibit dimerization of YycF, have bactericidal activity against *B. subtilis* and *S. aureus* (Gotoh et al., 2010).

Current knowledge in *B. subtilis, S. aureus* and *S. pneumoniae* indicates that this TCS maintains cell wall homeostasis, acting at the level of cell wall metabolism and/or cell wall remodeling, and cell division (reviewed in Jordan et al., 2008; Winkler and Hoch, 2008; Velikova et al., 2013). In *S. aureus* and *S. pneumoniae* YycFG also regulates genes involved in metabolism, stress response, virulence, multidrug resistance, host–microbe interactions and other regulatory pathways (Dubrac and Msadek, 2004; Mohedano et al., 2005; Howden et al., 2011; Delaune et al., 2012; Huang et al., 2013). There is also evidence that YycFG plays a role in membrane synthesis in several bacteria, thereby regulating membrane fluidity and permeability. In *S. aureus*, a point mutation in the *yycF* gene conferred temperature dependent sensitivity to several macrolide and lincosamide antibiotics, and also sensitivity to unsaturated long-chain fatty acids (Martin et al., 1999). In *B. subtilis*, depletion of the TCS resulted in decreased expression of the membrane phospholipid desaturase *des* gene, although this effect could have been indirect (Bisicchia et al., 2007), and in *S. pneumoniae* both induction and depletion of the TCS resulted in alteration of the transcription levels of genes encoding transport systems (Ng et al., 2003, 2005; Mohedano et al., 2005). We have also shown that overexpression of YycF altered expression of 12 *fab* genes, and increased levels of seven proteins involved in fatty acid biosynthesis. These alterations were accompanied by a change in the membrane fatty acid composition (Mohedano et al., 2005), similar to that detected by depletion of the FabT repressor of *fab* genes (Bisicchia et al., 2007). However, it is still unclear which form of YycF (phosphorylated or unphosphorylated) is responsible for this regulatory function. In *S. pneumoniae* the essentiality of YycF can be overcome by overexpression of the essential *pcsB* gene (Ng et al., 2003) and, in a strain (Pc-*pcsB+*) constitutively expressing the murein hydrolase PcsB (Bartual et al., 2014), deletion of YycF significantly affected expression of only 2 *fab* genes (*accD* and *fabZ*), in a chemically defined medium (Ng et al., 2005). However, this mutant required fatty acids for growth under some conditions, indicating that YycFG probably regulates membrane integrity.

Therefore, the YycFG system appears to play an important role in cell wall integrity in a wide range of Gram-positive bacteria, and may also modulate cell membrane integrity. The aim of this study was to gain a better understanding of the role of YycF in *S. pneumoniae*. We previously found evidence that, in addition to the phosphorylated form of YycF (P-YycF), the non-phosphorylated form (NP-YycF) may also have a role in control of gene expression in *S. pneumoniae* (Mohedano et al., 2005). To further elucidate the roles of P-YycF and NP-YycF species, we have now analyzed effects on transcription and protein expression during the course of TCS activation in the wild type strain (TIGR4[pPL101]) overproducing both YycF and YycG, and the YycG deficient mutant (TIGR4 *yycG*::*kan*[pPL100]) overproducing only YycF. Additionally, we have analyzed the interaction of the NP-YycF protein with the promoter region of the *FabTH*-*acp* operon, and its effect on the transcription of this operon.

## Materials and Methods

### Strains, Plasmids, and Growth Conditions

Strains used in this work are shown in **Table 1**. *S. pneumoniae* JNR7/87 (also denominated TIGR4, The Institute of Genomic Research website^1^) and derivatives were used for analysis of YycFG expression and *S. pneumoniae* R6 for cloning of *yycFG* genes. *Escherichia coli* M15[pREP4] strain was used for the cloning and expression of YycF.

**Table 1:** Strains used in this work.

Strain	Genotype and relevant features	Resistance	Reference
*S. pneumoniae* R61	Uncapsulated derivative of D39 strain.		Lacks, 1966
*S. pneumoniae* JNR7/87 (TIGR4)	Capsulated strain serotype 4.		Tettelin et al., 2001
*S. pneumoniae* TIGR4[pPL100]	Capsulated strain serotype 4. Maltose inducible YycF overexpressor.	EmR	Mohedano et al., 2005
*S. pneumoniae* TIGR4*yycG::kan*[pPL100]	Capsulated strain serotype 4 *yycG*::*kan* null mutant. Maltose inducible YycF overexpressor	Em^R^, Km	This work
*S. pneumoniae* TIGR4[pPL101]	Capsulated strain serotype 4.	Em^R^	This work
	Maltose inducible YycF and YycG overexpressor		
*E. coli* M15[pREP4]	F^-^, Φ80ΔlacM15, *thi, lac*^-^, *mtl*^-^, *recA*^+^. Expresses constitutively LacI repressor.	Km^R^	Invitrogen
*E. coli* M15[pREP4][pPL102]	F^-^, Φ80ΔlacM15, *thi, lac*^-^, *mtl*^-^, *recA*^+^. Inducible YycF overexpressor.	Amp^R^	This work

*S. pneumoniae* strains were grown at 37°C, without shaking, in AGCH medium (Lacks, 1966) supplemented with 0.2% yeast extract (AGCHY) and 0.8% sucrose (AGCHYS) or 0.8% maltose (AGCHYM). When required, erythromycin at 1 μg mL^-1^ and/or kanamycin at 250 μg mL^-1^ were added to the growth medium. The *S. pneumoniae* TIGR4 strain was cultivated in a Biosafety level 2 containment facility in line with health and safety guidelines.

*E. coli* strains were grown at 30°C with shaking in Luria broth. When required, ampicillin at 100 μg mL^-1^ and/or kanamycin at 25 μg mL^-1^ were added to the growth medium.

### Construction of the YycFG Mutants

The chromosomal *yycG* gene of the YycF overexpressor TIGR4[pPL100] strain (Mohedano et al., 2005) was interrupted by transformation using chromosomal DNA from *S. pneumoniae* R800 (*yycG*::*kan*) carrying the Km resistant cassette (Chastanet et al., 2001) inserted downstream of the 197 codon (nucleotide 592) of *yycG* (kindly provided by J.P. Claverys). Plasmid pPL101 was constructed as follows. The *yycFG* genes and their ribosomal binding sites were PCR amplified from the TIGR4 genome using the primers 5′-TTTGGTATAATAGCTAGCAAAAAGGTGAAC-3′ and 5′-AAAATACTGTATTGCTAGCCTATTTCACTC-3′, (the *Nhe*I restriction sites are underlined). The 2115 bp PCR product was then digested with *Nhe*I and cloned into the unique *Xba*I site of the plasmid pLS1RGFP expression vector (Nieto et al., 2000), between the pneumococcal P_M_ promoter and the *gfp* gene encoding the green fluorescent protein. The resulting plasmid pPL101 carries the transcriptional fusion P_M_-*yycFG-gfp* under control of the MalR repressor. The plasmid was established in the *S. pneumoniae* R6 strain by transformation, and transformants were selected by erythromycin resistance. The correct nucleotide sequence of the chromosomal insert of pPL101 was confirmed by DNA sequencing. Then, pPL101 was transferred to the *S. pneumoniae* TIGR4 capsulated strain by transformation and selection for erythromycin resistance.

Plasmid pPL102 was constructed as follows. The coding region of *yycF* was PCR amplified from the TIGR4 genome using the primers (5′-TTTGGTATAATAGTAGAGAAAAAGGATCCCATATG-3′ (a *Bam*HI site is underlined) and 5′-AATAAGAGGGTCACAACAAGCTTGAAACCTAA-3′, (a *Hind*III site is underlined). The 800 bp PCR product was digested with *Bam*HI and *Hind*III and cloned between the *Bam*HI and *Hind*III sites of pQE30 (Qiagen) to give rise to plasmid pPL102. The plasmid was established by electroporation in *E. coli* M15[pREP4] (plasmid encoding the lac repressor; Qiagen) and it carried the transcriptional fusion P_T5_-(His)_6_-*yycF* under control of the lac repressor. The correct nucleotide sequence of the chromosomal inserts of plasmids pPL101 and pPL102 as well as the *kan* chromosomal insert of TIGR4 *yycG*::*kan* were confirmed by DNA sequencing.

### Molecular Techniques

Standard procedures were used for PCR and cloning experiments. Genomic DNA was prepared as follows. Cultures of *S. pneumoniae* TIGR4 strain were grown to an absorbance of 0.7 at 650 nm. Samples of 1.5 mL of the cultures were sedimented and resuspended in 100 μL of a solution containing 730 mM sucrose, 150 mM sodium citrate, 2.3 mM sodium deoxycholate, SDS at 0.01% and 8 mg de RNasa I. Cells were lysed by incubation at 37°C for 15 min. Then, crude extracts were passed through a needle (25G 5/8 0,5 mm × 16 mm) to reduce their viscosity. Then, samples were deproteinated by two extractions with 100 μL of a mixture of phenol: chloroform (1:1 v/v) containing 4% isoamyl alcohol, DNA was precipitated in the presence of 0.3 M sodium acetate pH 7.0 with absolute ethanol and finally resuspended in 100 μL of 10:1 TE buffer (10 mM Tris pH 8.0 and 1 mM EDTA) and kept at -70°C. 1 μL of the extracts was used for PCR reactions.

Plasmid DNA was isolated from *E. coli* using the plasmid isolation kit from Roche, and from *S. pneumoniae* as previously described (Mohedano et al., 2005). Sequencing was performed using an ABI PRISM 320 sequencer (Perkin Elmer).

Competence and transformation procedures for the *S. pneumoniae* strain R6 were performed according to Lacks (1966), for the *S. pneumoniae* TIGR4 strain the synthetic competence-stimulating peptide (Håvarstein et al., 1996) at a concentration of 25 ng mL^-1^ was added to the transformation mixture. *E. coli* was transformed with plasmid by electroporation (Dower et al., 1988).

### Induction of Expression from P_M_ Promoter in *S. pneumoniae*

Uninduced cultures of pneumococcal strains were grown in the AGCHYS medium. Frozen cultures were diluted 1:1000 and grown overnight at 37°C until the cultures reached an A_650_ of 0.4. Overnight cultures were diluted 1:100 into fresh medium. The cultures were maintained in exponential growth phase by suitable dilution (at least twice for each experiment) with pre-warmed medium so that the cell density was always kept below an A_650_ of 0.4. Prior to induction, cells were grown to A_650_ of 0.4, harvested by centrifugation at 6,700 × *g* at room temperature, and resuspended in pre-warmed AGCHY and induction was carried out with 0.8% maltose for the times indicated in the Results. Long-term induction was performed by growth of the cultures in AGCHY medium containing 0.8% maltose plus 0.2% sucrose at an A_650_ of 0.4. Long-term induced cultures were obtained and kept in exponential growth phase as for uninduced cultures.

After growth, the cultures were harvested at 6,700 × *g* and washed twice with cold PBS buffer (10 mM Na_2_HPO_4_, 1 mM KH_2_PO_4_, 140 mM NaCl, 3 mM KCl) pH 8.0. Pellets were stored at -70°C for protein and RNA extraction.

### Measurement of GFP Expression by Fluorescence Microscopy

The pneumococcal cells present in maltose induced cultures were sedimented by centrifugation at 6,700 × *g* for 10 min, washed twice with PBS buffer (10 mM Na_2_HPO_4_, 1 mM KH_2_PO_4_, 140 mM NaCl and 3 mM KCl) pH 8.0 and resuspended in PBS buffer at the initial volume. Cells were directly analyzed, without fixing, by fluorescence microscopy using a Zeiss Axioplan Universal microscope using excitation standard FITC set D480/30 and emission TBP 460/530/610 filters.

### RNA Preparation and Transcriptional Analysis

RNA was prepared from 10 mL cultures following the QIAGEN RNeasy Midi Kit (QIAGEN) procedure. The total RNA concentrations were determined by UV spectrophotometry. The RNAs were checked for integrity and yield by analysis in 1% agarose gel. In addition, the quality and quantity of the RNA samples were checked using an RNA 6000 Nano assay with the Agilent 2100 Bioanalyzer (Agilent Technologies) according to the protocol of the manufacturer.

For transcriptional analysis *fab* and *yycFG* gene-specific probes for *S. pneumoniae* TIGR4 strain were used, made as previously described (Mohedano et al., 2005).

Fluorescent labeling of RNA and hybridization were performed as previously described (Mohedano et al., 2005). Analysis was conducted on RNA purified from three independent cultures of each strain. The standard deviation of the mean value of the fold increase or decrease obtained in each hybridization for each induction condition (compared to the TIGR4[pLS1RGFP] control strain grown in the same condition) was calculated and these data are shown in **Figure 2A**.

### Preparation of Protein Extracts

For the preparation of membrane vesicles and cytosolic protein fractions, 150 mL cultures of *S. pneumoniae* TIGR4[pPL101] and TIGR4[pLS1RGFP] were challenged during 45 min with maltose. Then, cultures were sedimented by centrifugation, washed with 50 mL of PBS buffer pH 8.0 and resuspended in 15 mL of a solution containing 100 mM HEPES (pH 8), 10% glycerol, 1 mM MgSO_4_, 150 mM NaCl, and Benzonase (250 U μL^-1^; Boehringer Mannheim). Afterward, the cells were disrupted by one passage through a French press at 12,000 lb in^-2^. The samples were centrifuged for 20 min at 3,000 × *g* at 4°C. The resulting supernatant was subjected to ultracentrifugation at 150,000 × *g* at 4°C for 1 h. Then, the supernatant (designated cytosolic protein fraction) was used for the YycG auto-phosphorylation reaction and western blot detection of YycF. Membrane vesicle preparations were obtained by resuspension of the pellet in 200 μL of 50 mM Tris-HCl (pH 7.5), 50 mM KCl, 5 mM MgCl_2_, 1 mM dithiothreitol (DTT), yielding a protein concentration of about 18 mg mL^-1^.

For 2D gel analysis, total protein extracts were prepared from frozen pellets from 100 mL induced cultures as previously described (Mohedano et al., 2005). The total protein concentration in the extracts was quantified after separation of the proteins on 12.5% SDS-polyacrylamide gels and staining the resulting bands with 0.25% Coomassie Brilliant Blue. The quantification was performed with the program Quantity One 4.2.1 in a Gel Doc 2000 Molecular Analyst (Bio-Rad Laboratories). The Mark 12 Unstained Standard (Invitrogen) was used as reference.

### Proteomic Analysis

Bacterial lysates containing 100 μg of protein were analyzed in individual experiments as previously described (Mohedano et al., 2005). Briefly, the proteins were separated in the first dimension using gel strips pH 4.0–7.0 and in the second dimension on 10% duracryl gels. Gels were stained with Sypro Ruby. The proXPRESS Proteomic Imaging System (Perkin Elmer) was used for the imaging. The protein spots present in the 2D-gels were quantified with the PDQuest 2D analysis 7.1.0 program (Bio-Rad Laboratories). 400 stained spots were matched in all gels and used for normalization of the average intensity. The spots of interest were digested with trypsin and analyzed by the John Innes Centre Protein Sequencing facility using matrix-assisted laser desorption ionization, -time of flight mass spectrometry (MALDI-TOF-MS). Peptide peak lists were searched against the databases of *S. pneumoniae*^2^.

### Detection of Autophosphorylation of YycG in Membrane Vesicles

YycG autophosphorylation assays were performed basically as previously described (Wagner et al., 2002). Briefly, reactions were performed in a buffer containing 50 mM Tris-HCl (pH 7.5), 50 mM KCl, 5 mM MgCl_2_, 1 mM DTT, 0.33 mM NADH, 2.5 mM phosphoenolpyruvate, 1 mM ATP, 0.165 U of pyruvate kinase, 0.25 U of lactate dehydrogenase (from rabbit muscle; Sigma), 15 μCi of [γ-^32^P]ATP, 3.8 mM *para*-nitrophenyl phosphate, 0.5 mM O-phospho-L-threonine, 5 mM O-phospho-L-serine, 50 mM sodium orthovanadate, and 80 μg of membrane vesicles or of preparations of cytosolic proteins. After incubation at 30°C for 10 min, the reaction was stopped by the addition of SDS loading buffer containing at final concentration 50 mM DTT, 4% SDS, 50 mM Tris-HCl (pH 6.8), 12.5% glycerol, 2.5 mM NaH_2_PO_4_, 0.05% bromophenol blue, and 5 mM EDTA. Samples were analyzed by 10–20% PAA-SDS and phosphorylated proteins were revealed by autoradiography using Kodak Biomax MS films. SeeBlue Plus2 (Invitrogen) pre-stained protein standard was also run in the gel to estimate the molecular weight of the phosphorylated proteins.

### Western Blot Detection of YycF

For the detection of YycF, preparations of total proteins, cytosolic proteins and membrane vesicles were fractionated by 10–15% SDS-PAA. SeeBlue Plus2 (Invitrogen) pre-stained protein standard was also run in the gel to estimate the molecular weight of the blotted proteins. The separated proteins were electro-blotted onto a nitrocellulose membrane (Schleicher & Schuell BA85 0,45 μm) by use of a Mini trans-blot electrophoretic transfer cell (Bio-Rad). Hybridization and blotting were performed according to the protocol described by Zilhao et al. (1996). The antibodies, obtained by rabbit immunization with purified YycF, were used at a dilution of 1:300.000. Rabbit anti-IgG conjugated with alkaline phosphatase (Sigma) at a dilution 1:30.000 was used as a secondary antibody. Conditions of hybridization were performed according to the protocol of Immun-Star Chemiluminescent Protein Detection System (Bio-Rad).

### Expression and Purification of Recombinant YycF

The expression of (His)_6_-*yycF* was induced in *E. coli* M15[pREP4][pPL102] cells grown in LB at 30°C, when cultures reached an A_600_ of 0.5, by addition of 1 mM IPTG and further incubation at 30°C. Production of (His)_6_-YycF fusion protein increased up to 4 h after induction (Supplementary Figure 1A). However, analysis of the soluble and insoluble protein fractions revealed that the regulator became partially insoluble, with a maximum yield of soluble YycF at 2 h after induction (Supplementary Figure 1B). This condition was used to overproduce the response regulator from 500 mL of bacterial cultures. After induction for 2 h, cells were harvested by centrifugation and resuspended in ice-cold buffer A containing 50 mM Tris-HCl (pH 7.4) and 500 mM NaCl. Crude extracts were prepared by passing the cells three times through a French pressure cell at 12,000 lb min^-2^. Cellular debris was removed by centrifugation at 10,600 × *g* for 40 min at 4°C. Nucleic acids present in the supernatant were removed by treatment with 0.2% polyethylenimine for 30 min at 4°C and ultracentrifugation at 67,500 × *g* for 30 min at 4°C. YycF was precipitated from the supernatant with 70% ammonium sulfate (1 h at 4°C). The precipitate was recovered by centrifugation at 10,600 × *g* for 20 min at 4°C, resuspended in 20 mL of buffer A, dialyzed overnight against buffer A to remove ammonium sulfate and ultracentrifuged at 67,500 × *g* for 40 min at 4°C to remove insoluble particles. Then, 20 mM imidazole was added to the supernatant prior to YycF purification by nickel-affinity chromatography (5 mL HisTrap HP column, Amersham Biosciences) using the HPLC ÄKTA purifying system (Amersham Biosciences). After loading the sample, the column was washed with buffer A plus 20 mM imidazol (500 mL) at 0.5 mL min^1^ and a 20 mM–1.0 M linear imidazole gradient (120 mL) was applied at 1 mL min^-1^. YycF eluted at approximately 200 mM imidazole (Supplementary Figure 2). Fractions containing the protein were dialyzed against buffer A to remove imidazole and YycF was concentrated using Amicon ultra 10 K devices (Millipore) to its highest soluble concentration (2 mg mL^-1^).

### Determination of Copy Number of YycF

Total protein extracts prepared from 30-fold concentrated cultures induced for 10 min and standards of increasing concentrations of purified (His)_6_-YycF (from 2.5 ng to 10 ng in Supplementary Figure 3A and from 0.05 μg to 1 μg in Supplementary Figure 3B) were fractionated and subjected to Western blot analysis as detailed above. YycF bands from TIGR4[pLS1RGFP] (5.76 μL), TIGR4*yycG*::*kan*[pPL100] (5.75 μL) and TIGR4[pPL101] (5.8 μL) protein extracts and from the purified protein were detected with a chemo-luminescent detector (“Intelligent Darkbox” LAS-3000, Fujifilm) and quantified with the Image Reader LAS-3000 software. The analysis revealed a concentration of YycF in the original cultures of 25, 463, and 344 ng mL^-1^, for the control strain and YycFG and YycF overexpressors, respectively.

The number of YycF molecules per mL of culture was calculated using the Avogadro number and taking into account the molecular mass of YycF (26,815 Da). Plate counting of the induced cultures revealed, respectively, values of 9.91 × 10^7^ CFU mL^-1^, 1.41 × 10^8^ CFU mL^-1^ and 2.07 × 10^8^ CFU mL^-1^. Analysis of the cultures by microscopy as described above showed an average cell number per chain, which should correspond to a colony forming unit value of 10, 5 and 3.5 (see Supplementary Figure 4). Thus, the estimated copy number of YycF is 565, 14,740 and 10,685 for the control, YycFG and YycF overexpressing strains, respectively.

### Analytical Ultracentrifugation Assays

Sedimentation equilibrium was performed to determine the state of association of the P-YycF and NP-YycF. The experiments, carried out using from 5 to 20 μM protein, were performed at 20°C using different speeds (12,000 and 15,000 rpm) and wavelengths (277, 240, and 235 nm) with short columns (80–100 μL) in an XL-A analytical ultracentrifuge (Beckman) equipped with UV-visible optics detection system, using an An50Ti rotor and 12-mm double sector or six-hole Eponcharcoal centerpieces. All samples were in a buffer containing 50 mM Tris-HCl pH 7.4 and 500 mM NaCl. After the equilibrium scans, a high speed centrifugation run (50,000 rpm) was done to estimate the corresponding base-line offsets. Weight average buoyant molar masses of the proteins were determined by fitting data to a single species model using the HeteroAnalysis program (Cole, 2004). The reported errors of the fit of the molar masses correspond to two S.D. values (95% confidence limits). The corresponding protein molar masses were determined from the experimental buoyant masses using 0.738 mL g^-1^ as the partial specific volume of YycF (calculated from the amino acid composition using the SEDNTERP program; Laue et al., 1992). For sedimentation velocity analysis the runs were carried out at 50,000 rpm and 20°C and the sedimentation profiles were registered every 5 min at 277 nm. Sedimentation coefficient distributions were calculated by modeling of sedimentation velocity data using the *c(s)* method (Schuck, 2000), as implemented in the SEDFIT program, from which the corresponding sedimentation coefficients (*s*-values) were obtained.

### Chemical Phosphorylation of YycF and Analysis

The phosphorylation reaction was performed basically as described previously (Ng et al., 2005). Briefly, 5 μM of YycF was incubated with 10 mM of acetyl phosphate in 100 μL reaction containing 75 mM NaCl, 50 mM Tris pH 6.0 and 20 mM MgCl_2_, at 37°C for 1 h. The extent of phosphorylation was determined by analytical reverse-phase HPLC at 25°C on a Phenomenex Jupiter 5u 300A C4 column (250mm × 4.6 mm) attached to an AKTA basic 10 HPLC system from Amersham Bioscience/GE Healthcare with a conductivity monitor pH/C-900 (Amersham Bioscience/GE Healthcare). Mobile phases A (20% acetonitrile, 0.1% trifluoroacetic acid) and B (60% acetonitrile, 0.1% trifluoroacetic acid) were mixed to form the gradient 80% phase A-20% phase B to 0% phase A-100% phase B in 40 min, at a flow rate of 1 mL per min. Polypeptides were detected by monitoring absorbance at 220 nm.

### Electrophoretic Mobility Shift Assays

For these experiments the DNA samples used were the A (194 bp) and B (318 bp) amplicons including, respectively, the *fabT* and the *malX* promoter regions. The radiolabeled amplicon A and the unlabeled B amplicon were generated by PCR by use of the following oligonucleotides: 5′-CATTCGGAGAGAAGAAGACCTAAATTTA-3′ and 5′- CCTCAATTACAAGGACATTGTTAAATATAGAT-3′ for amplicon A and 5′-GATTTTAAATTTTTTATGGATTACTGTT-3′ and AGAAACCATTACTGTACTTAATAAA for amplicon B, 150 μM [α-^32^P]dCTP (3000 Ci mmol^1^, 1mCi mL^-1^) and 150 μM of each dATP, dTTP and dGTP for the A amplicon or four unlabeled dNTPs for amplicon B and 2 U of Pfu DNA polymerase (Stratagene). The amplicons were purified using 5% polyacrylamide gel prior to their use for binding reactions, which were performed in 20 μL final volume of 50 mM Tris-HCl (pH 7.8), 500 mM NaCl and 4 mM MgCl_2_. In some cases 25 ng μL^-1^ of poly-(dI-dC) or amplicon B at 1 nM were added to the reaction as unspecific competitor DNAs. Labeled DNA fragments (0.5–1.0 nM) and YycF (1.0–5.0 μM) were incubated at 0°C for 30 min. The reactions were loaded onto a 5% non-denaturing polyacrylamide gel and run with TBE buffer for 2.5 h at 100 V and 4°C. After completion of electrophoresis, the gels were dried, autoradiographed, and the bands were detected by use of a Phosphorimager GS800 (Fujifilm).

### *In vitro* transcription

Transcription reactions were performed at 37°C for 15 min in a 25 μL volume containing 40 mM Tris-HCl (pH 7.4), 10 mM MgCl_2_, 290 mM NaCl, 0.1 mM EDTA, 300 μM of each of ATP, GTP and CTP, 100 μM UTP, 0.25 μM [α-^32^P]UTP (800Ci mmol^1^; 10 μCi μL^1^), 1 nM template DNA, 0.05 μg μL^-1^ BSA, and 0.22 U (20 nM) of RNA polymerase σ^70^ holoenzyme. To test the effect of YycF in transcription the reaction was performed after binding of the DNA with different concentrations of YycF (0.25–10 μM). Binding reactions were performed in a 20 μL volume containing 50 mM Tris-HCl (pH7.4), 364 mM NaCl, 2 mM MgCl_2_, 0.125 mM EDTA, and 1.25 nM DNA (either unlabelled A or B amplicons). After 30 min of incubation at room temperature, transcription was started by the addition of 5 μL of nucleoside triphosphate mix containing the RNA polymerase and the appropriate amount of MgCl_2_. Reactions were terminated by the addition of an equal volume of 100 mM EDTA containing 2% SDS. Samples were filtered through MicroSpin G-25 columns (GE Healthcare) to remove the unincorporated nucleotide and precipitated by addition of 10 μg of tRNA, sodium acetate (pH 7), and 3 volumes of ethanol. Pellets were suspended in RNA loading buffer (80% deionized formamide, 1X Tris-borate-EDTA, 0.025% bromophenol blue, 0.025% xylene cyanol) and analyzed on a 8% polyacrylamide-urea gel followed by autoradiography. The molecular weight standard was the A+G Maxam and Gilbert sequencing reaction of a 174-bp amplicon generated by PCR using the 5′-^32^P-labeled oligonucleotides 5′-TATGGAAGCAACCACGCT-3′ and 5′-TCAGCATAACTGAGC C-3′.

## Results

### Construction and Analysis of the YycFG TCS Mutants

To analyze a potential role of the phosphorylated and non-phosphorylated forms of YycF in fatty acid biosynthesis, the TIGR4[pPL101] and TIGR4 *yycG*::*kan*[pPL100] strains were constructed. The TIGR4[pPL101], containing the wild-type chromosomal *yycFG* operon and the P_M_-*yycFG*-*gfp* transcriptional fusion, provides the background to test the influence of overexpression of the whole YycFG system. In this strain, the YycF is predicted to be phosphorylated due to the concomitant over production of YycG. By contrast, the TIGR4 *yycG*::*kan*[pPL100] strain, which harbors the plasmidic P_M_-*yycF*-*gfp* transcriptional fusion and carries a knockout *yycG* gene in its chromosome, allows the testing of overproduction of YycF in the absence of YycG and presumably with residual/low phosphorylation. Conditions for induction of the P_M_ promoter with maltose were standardized for both strains by fluorescence microscopy and quantification of GFP expression by fluorescent spectroscopy (results not shown). Transient induction of the P_M_ promoter and expression of *gfp* was achieved by transfer of the cultures from AGCHY medium containing 0.8% sucrose to AGCHY medium containing 0.8% maltose. Transient induction for 30 min did not affect the growth rate of any of the strains tested (doubling time of 31±2 min), but after 60 min anomalous cell morphology was detected in the overproducing strains (TIGR4[pPL100], TIGR4[pPL101] and TIGR4 *yycG*::*kan*[pPL100]) but not in the TIGR4[pLS1RGFP] control strain (Supplementary Figure 4) indicating that the deregulation of YycFG expression leads to aberrant control of cell division. As previously reported for YycF (Mohedano et al., 2005), long term overexpression of YycFG was only feasible in defined medium containing 0.8% maltose plus 0.2% sucrose (AGCHYMS), which only partially induced expression of the TCS from the plasmid P_M_ promoter, and osmotically stabilized the cells. Under these conditions, the TIGR4 wild type strain overexpressing YycF or YycFG showed only slightly slower growth (doubling times of 32±5 and 40±6 min, respectively) compared to the TIGR4[pLS1RGFP] control strain (doubling time of 23±2 min). However, the TIGR4 *yycG*::*kan*[pPL100] mutant strain was unable to grow in the AGCHYMS medium, indicating that, in the absence of a functional YycG, the overexpresion of YycF is deleterious to the cells.

**FIGURE 1:**
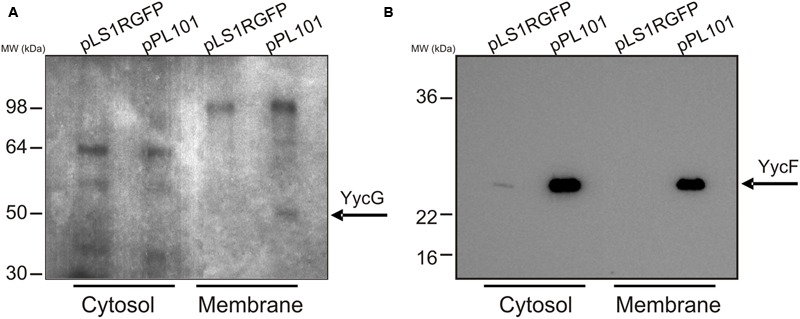
**Detection of YycG and YycF in membrane and cytosolic protein fractions.** Cultures of TIGR4 carrying either pLS1RGFP or pPL101 plasmids were induced and then, membrane and cytosolic protein extracts were prepared. **(A)** the extracts were [^32^P]–phosphorylated *in vitro*, fractionated by SDS-PAGE, and protein bands detected by autoradiography. **(B)** the extracts were fractionated by SDS-PAGE followed by western blotting and detection using antibody developed against YycF (see details in Materials and Methods). Position of migration of the SeeBlue Plus2 (Invitrogen) pre-stained protein standard are indicated. Phosphorylase B, 148 kDa; bovine serum albumin, 98 kDa; glutamic dehydrogenase, 64 kDa; alcohol dehydrogenase, 50 kDa; carbonic anhydrase 36 kDa; myoglobin red 22 kDa and lysozyme 16 kDa.

The pneumococcal YycG protein contains a transmembrane segment at its N-terminus, and Wayne et al. (2010) detected *in vivo* YycG randomly distributed in the membrane of dividing *S. pneumoniae* serotype 2 cells. Overproduction of YycG in TIGR4[pPL101] could result in the production of a cytosolic defective protein, unable to perform auto-phosphorylation. To investigate this possibility, crude protein extracts from the cytosolic fraction and membrane vesicles were prepared from TIGR4[pLS1RGFP] and TIGR4[pPL101] cultures, induced with maltose for 30 min and tested using an *in vitro* [^32^P]-phosphorylation assay. Analysis by SDS-PAGE (**Figure 1A**) revealed several bands corresponding to phosphorylated proteins in all four extracts. However, a band of approximately 50 kDa (predicted molecular mass of YycG = 51.7 kDa) was detected only in the membrane preparation of the YycFG over-expressing strain, suggesting that a functional membrane associated YycG was indeed overproduced. The presence of YycF in the protein extracts of these strains was tested by Western blotting using antibodies raised against purified recombinant YycF (**Figure 1B**). The regulator YycF was detected in the cytosolic fraction of both TIGR4[pLS1RGFP] (control) and TIGR4[pPL101] (overexpressing YycFG) strains, as previously observed in *S. pneumoniae* serotype 2 (Wayne et al., 2010) and, as expected, the amount of YycF was higher in the latter strain. In addition, the protein was detected in the membrane fraction of the TIGR4[pPL101] strain, probably because YycF was associated with YycG.

### Transcriptional and Proteomic Analysis of the YycFG TCS Mutants

To study the effect of YycFG and YycF overproduction on pneumococcal physiology, we performed a transcriptional and proteomic analysis of TIGR4 *yycG*::*kan*[pPL100], TIGR4[pPL101] and the control strain TIGR4[pLS1RGFP]. The three strains were transiently induced with maltose during 10 and 30 min and then harvested for RNA and protein extraction. TIGR4[pPL101] and the control strain were also tested by protein analysis after long-term maltose-induction of the expression plasmids.

**FIGURE 2:**
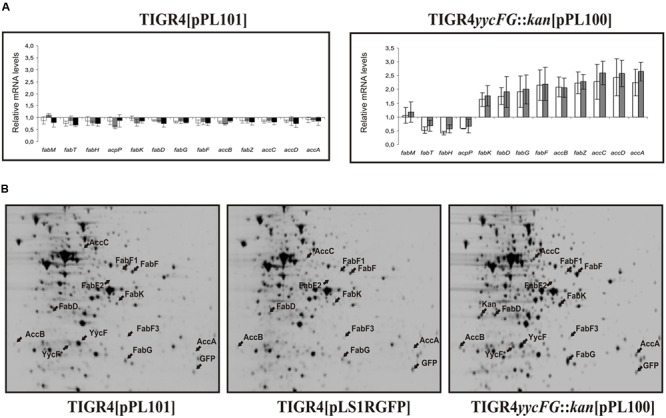
**Transcriptional and proteomic analysis of the influence of YycFG induction on fatty acid biosynthesis in *S. pneumoniae* strains.** Cultures of TIGR4[pLS1RGFP] (control strain), TIGR4 *yycG*::*kan*[pPL100] and TIGR4[pPL101] were induced with maltose. **(A)** shows the mRNA levels of fatty acid biosynthesis genes in strains TIGR4[pPL101] (left panel) and TIGR4 *yycG*::*kan*[pPL100] (right panel) relative to the control strain after 10 min (white bars), 30 min (gray bars) and long-term (black bars) induction. The length of the error bars represents the standard deviations on either side of the means. **(B)** shows the 2D gel electrophoretic analyses of total protein extracts of each strain after 30 min induction. Protein spots corresponding to the proteins listed in **Table 2** and the kanamycin resistance (kan) protein are indicated by arrows.

Transcription analysis revealed that the expression of genes involved in fatty acid biosynthesis (Sp0417-Sp0427) was affected in the TIGR4 *yycG*::*kan*[pPL100] strain overproducing YycF, but not in the TIGR4[pPL101] strain overproducing the whole system. As shown in **Figure 2A**, after 10 or 30 min of maltose induction *fabT, fabH* and *acpP* transcripts were repressed in TIGR4 *yycG*::*kan*[pPL100] while the remainder were significantly enhanced.

The proteomic analysis showed that several proteins were differentially expressed in one or in both overproducer strains, which were further identified by tryptic digestion and MALDI–TOF mass spectrometry (Supplementary Table S1 and summarized in **Table 2**). As expected, an increased amount of the YycF protein was detected in both overproducer strains at all times post induction. Two protein spots corresponding to the response regulator were identified (YycF^∗^ and YycF in **Figure 2B**). The more positively charged spot YycF was more abundant than YycF^∗^. In addition to the difference in the isoelectric point, they were also different in their apparent molecular mass, 24.3 kDa for YycF (the expected molecular mass for the response regulator) and 23.2 kDa for YycF^∗^. MALDI–TOF mass spectrometry indicated that YycF^∗^ lacks the carboxy-terminal region of YycF (results not shown). Therefore, YycF^∗^ probably corresponds to a proteolytic fragment of the RR, instead of its phosphorylated form as previously proposed (Mohedano et al., 2005).

**Table 2:** Proteins affected by YycF induction, as detected by proteomic analysis.

Spot	Ratio of induction^1^							pI	MW (kDa)
	TIGR4 *yycG*::*kan*[pPL100] TIGR4[pLS1RGFP]		TIGR4 y*ycG*::*kan*[pPL100] TIGR4[pPL101]		TIGR4[pPL101] TIGR4[pPLS1RGFP]				
	10 min	30 min	10 min	30 min	10 min	30 min	Long-term		
FabK	2.2	2.2	2.1	2.2	1.1	1.0	1.1	5.12	34.156
FabD	2.0	2.8	1.8	2.6	1.1	1.1	1.0	4.45	33.184
FabG	2.5	2.8	2.2	2.6	1.1	1.1	1.0	5.34	25.739
FabF^2^	2.0	2.3	1.8	2.3	1.1	1.0	1.0	5.45	44.075
FabF1^2^	3.5	2.8	2.6	2.6	1.4	1.1	0.7	5.29	45.157
FabF2^2^	1.6	2.7	1.5	2.2	1.1	1.3	1.6	5.15	42.875
FabF3^2^	2.0	2.4	2.0	1.8	1.0	1.3	1.3	5.32	26.699
AccB	1.9	2.0	1.7	2.0	1.1	1.0	1.2	4.17	17.023
AccC	1.9	2.6	2.5	2.2	0.8	1.1	1.2	4.85	49.793
AccA	3.0	2.1	2.3	2.4	1.3	0.9	0.9	6.31	28.230
YycF^3^	>164	>20.6	0.9	0.8	>182	>25.2	>32.7	4.78	24.265
YycF^3^	>454	>68.4	1.1	0.8	>413	>58.6	>74.8	4.71	23.251

*AccA, acetyl-CoA carboxylase carboxyl transferase alpha subunit; AccB, acetyl-CoA carboxylase biotin carboxyl carrier protein; AccC, acetyl-CoA carboxylase biotin carboxylase; FabD, Malonyl CoA-acyl carrier protein transacylase; FabF, Oxoacyl-(acyl-carrier-protein) synthase II; FabG, 3-oxoacyl-(acyl-carrier-protein) reductase; FabK, Enoyl-(acyl-carrier-protein) reductase.*

*^1^The ratios were calculated by dividing the mean values of the spot intensities (from three independent experiments) obtained for TIGR4 yycG::kan*[pPL100]*, TIGR4[pPL101] and TIGR4[pPL100] and TIGR4[pLS1RGFP]. The mean values and the standard deviation of the spot intensities are depicted in Supplementary Table S1.*

*^2^Different isoforms of FabF.*

*^3^Different isoforms of YycF.*

The inability to detect YycF and YycF^∗^ in the extracts of TIGR4[pLS1RGFP] made it difficult to determine the increase of YycF levels in the overexpressing strains compared to the control strain. However, Western blot analysis of protein extracts after 10 min of induction (using purified YycF as the standard for protein concentration), together with the determination of the number of bacterial cells used for the preparations, established the copy number of YycF in the three strains (see Materials and Methods and Supplementary Figure 3). This analysis revealed that there were approximately 565 YycF molecules per cell in the control strain, and approximately 14,740 and 10,685 YycF molecules per cell in TIGR4[pPL101] (26.1-fold induction) and TIGR4 *yycG*::*kan*[pPL100] (18.9-fold induction) respectively. These results correlate with the transcriptional analysis in which 32.5- and 22.9-fold induction of the regulator expression were detected in the YycFG and YycF overexpressing strains, respectively (results not shown).

In the 2D-gel proteomic analysis, an additional major spot was present only in protein extracts from TIGR4 *yycG*::*kan*[pPL100] which was the product of the *kan* gene present in the chromosome of this strain.

**FIGURE 3:**
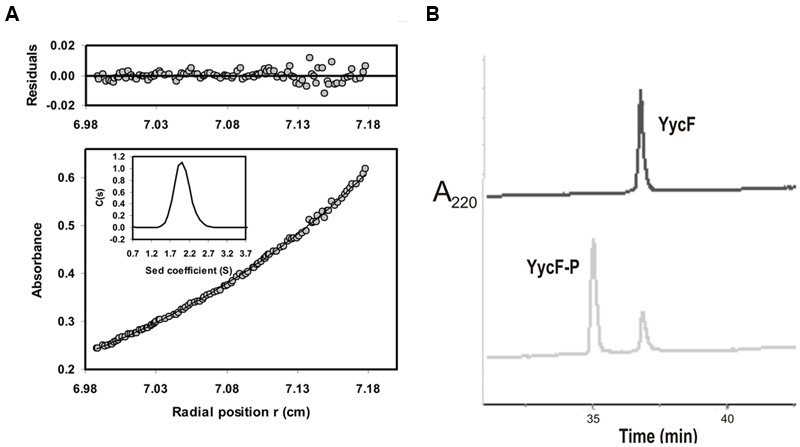
**Physicochemical analysis of YycF.** Analytical ultracentrifugation profile of purified YycF **(A)**. The sedimentation equilibrium profile of 20 μM YycF at 15,000 rpm, 20°C, measured at wavelength of 277 nm. Gray circles represent the experimental data; the continuous line is the best fit distribution for a single species at sedimentation equilibrium. The average molecular weight was 27,700 Da at this protein concentration. The residuals to the fit are shown in the upper part of the figure. Insert: Sedimentation velocity (50,000 rpm, 20°C) distributions of the same YycF preparation shown in the main figure. Chromatographic analysis of P-YycF and NP-YycF **(B)**. The figure depicts the elution profile (detected by absorbance at 220 nm) of purified YycF after fractionation using a C4-HPLC column prior to (upper profile), or after (lower profile), phosphorylation with acetyl phosphate (see details in Materials and Methods).

Ten polypeptides, whose levels were increased only in TIGR4 *yycG*::*kan*[pPL100], were identified as proteins involved in fatty acid biosynthesis (AccA, AccB, AccC, FabK, FabD, FabG and four isoforms of FabF). A similar increase in the levels of these proteins was previously detected in the proteomic analysis of TIGR4[pPL100] (Mohedano et al., 2005), in which only the most abundant isoform of FabF (designated FabF in **Figure 3B**) was detected. The apparent molecular mass of FabF was of 44.1 kDa which would correspond to FabF oxoacyl-(acyl-carrier-protein) synthase II. Two spots, FabF3 and FabF4, showed lower apparent molecular masses (42.9 and 26.7 kDa) and were probably proteolytic fragments of FabF. However, FabF2 (45.2 kDa) seems to be a post-transcriptionally modified form of FabF. These proteomic results agree perfectly with the transcriptomic data, where transcription of genes encoding these seven proteins was also increased in the TIGR4 *yycG*::*kan*[pPL100] strain.

### The Interaction of NP-YycF with DNA

The above results indicated that the non-phosphorylated form of YycF regulates the fatty acid biosynthesis in *S. pneumoniae* by regulation of *fab* genes expression. To confirm this hypothesis, the RR was purified in order to carry out DNA-protein interaction studies. Previous studies indicated that the *S. pneumoniae* YycF was mostly insoluble when produced in *E. coli* (Clausen et al., 2003), though it can be overproduced as a soluble (His)_6_-YycF fused protein (Ng et al., 2005). Therefore, the pPL102 plasmid encoding a (His)_6_-YycF was constructed and the protein was overproduced in *E. coli* and purified by affinity chromatography with a yield of 4 mg per liter of culture (see Materials and Methods). The solubility of the protein was enhanced at higher ionic strengths and the maximum concentration of soluble protein ranged from 0.8 to 1 mg mL^-1^ (28.6–35.8 μM) in 0.5 M NaCl. It is known that YycF acts as a dimer in other bacteria and to explore the multimerization state of the pneumococcal protein, analytical ultracentrifugation analyses were conducted using three concentrations of YycF (5, 10, and 20 μM). The results indicated a mass of 26,289 Da that essentially corresponds to that of the protein monomer (27,920 Da). **Figure 3A** shows the experimental gradient obtained for the 20 μM (0.56 mg mL^-1^) sample and the corresponding best fit gradient (solid line). Furthermore, the sedimentation velocity analysis indicated that YycF is a monodisperse protein in solution under the test conditions, and the sedimentation coefficient (S) obtained was compatible with the monomeric form of the protein (data not shown). Therefore, under the conditions tested, the purified pneumococcal YycF was a monomeric protein.

To generate a standard sample of P-YycF, the protein was chemically phosphorylated with acetyl phosphate as previously described (Ng et al., 2005) and its phosphorylation state was analyzed by HPLC. Two peaks were detected by HPLC (**Figure 3B**), a minor one that has the same elution time as the control NP-YycF, and a major peak that ranged from 70 to 90 % of the total protein concentration. However, analytical ultracentrifugation showed that most of the P-YycF was insoluble (results not shown). Attempts to phosphorylate in the presence of 0.5 M NaCl to increase solubility were unsuccessful. Consequently, it was not possible to test P-YycF in protein-DNA interaction studies. However, the HPLC analysis of the purified soluble YycF overproduced in *E. coli* revealed only one peak corresponding to the non-phosphorylated form, thus supporting the homogeneity of the protein preparation and suggesting that the RR was not phosphorylated *in vivo* in *E. coli* as reported for other response regulators (Cybulski et al., 2004). Therefore, the purified NP-YycF was used to analyze its role in controlling the transcription of *fab* genes.

**FIGURE 4:**
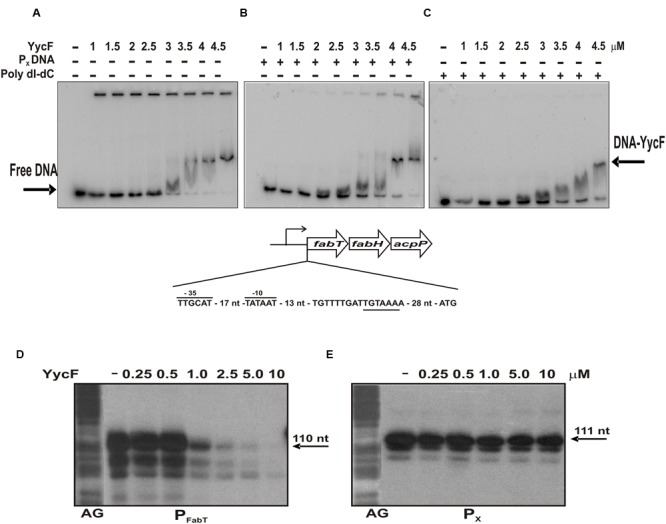
**Regulation of FabT expression by YycF.** Upper panels, detection of the binding of YycF to the P_FabT_ promoter region. Autoradiograms of gel shift assays using the amplicon A and increasing concentrations of YycF in absence **(A)** or in presence of either poly (dI-dC) **(B)** or amplicon B **(C)**. Arrows indicate the position of the retarded complex and free DNA. A schematic representation of the promoter region of *fabTH-acpP* operon including the -35 and -10 boxes of its putative promoter (P_FabT_) as well as the putative binding site of YycF (underlined) is also depicted. Lower panels, effect of YycF on *in vitro* transcription. The run off transcripts obtained from P_FabT_ **(D)** and from P_X_ **(E)** synthesized by the *E. coli* RNA polymerase in the absence or in the presence of the indicated concentrations of YycF are depicted. The position and length of the transcripts generated are indicated. On the left of each panel is a G + A sequence ladder generated with an unrelated DNA fragment.

FabT regulator is a repressor of *fab* genes expression (Lu and Rock, 2006) and we have previously proposed that NP-YycF would induce transcription of the *fabKDGF-accB-fabZ-accCDA* operon by repression of expression of the *fabTH-acpP* operon (Mohedano et al., 2005). To test this hypothesis, we analyzed the ability of YycF to bind the 194 nt amplicon A, which includes the *fabTH-acpP* promoter (P_FabT_) region. The experiments were performed in the presence of 0.5 M NaCl to ensure that YycF was in a soluble and monomeric form. Band-shift experiments revealed that the NP-YycF bound to the promoter region, generating one DNA-YycF complex (**Figure 4A**). The binding of the RR seemed to be specific, since the complex was also detected in the presence of non-specific competitors: (i) a DNA fragment of 318 nt in length containing the pneumococcal P_X_ promoter (**Figure 4B**), which has a A+T content similar to the FabT promoter or (ii) poly (dI-dC; **Figure 4C**). In addition, no YycF binding was observed when using only the radiolabeled P_X_ promoter region (results not shown).

We next investigate whether this specific binding indeed regulates transcription from the P_FabT_ promoter (**Figure 4D**). The P_FabT_ has a canonical extended -10 (TGATAAT) region for binding of the vegetative σ factor of bacterial RNA polymerases (RNAP). Therefore, it was predicted that the *E. coli* RNAP could utilize this promoter. Thus, the same DNA fragment used for band shift experiments was used for run-off *in vitro* transcription experiments performed with the *E. coli* RNAP. As anticipated, the enzyme was able to synthesize the expected 110 nt transcript from the FabT promoter (**Figure 4D**). Moreover, the presence of YycF in the reaction resulted in the inhibition of transcription in a dose-dependent mode. This inhibition was specific for the *fabT* promoter region, since the presence of YycF did not affect transcription from the pneumococcal P_X_ promoter by the RNAP (**Figure 4E**), which is a constitutive promoter in the absence of the MalR repressor (Nieto et al., 2001).

Collectively, these data indicate that the non-phosphorylated form of YycF regulates transcription of fatty acid biosynthesis genes in *S. pneumoniae*. Direct binding of the pneumococcal enzyme to the promoter region of FabT repressor inhibits *fabT* synthesis and, consequently, increases transcription of the *fabKDGF-accB-fabZ-accCDA* operon.

## Discussion

In this work, we have provided evidence that, in *S. pneumoniae*, non-phosphorylated-YycF is involved in regulation of membrane biogenesis. The transcriptional and proteomic analyses presented here showed that overexpression of YycF in the absence of YycG (but not in the overexpression of both) resulted in alterations of the expression of proteins involved in fatty acid biosynthesis. In TIGR4 *yycG*::*kan*[pPL100], a decrease in transcription of the *fabTH-acpP* operon was accompanied by an increase in transcription of the *fabKDGF-accB-fabZ-accCDA* operon and higher levels of some of its encoded proteins. The FabK, FabG, FabZ, and FabF proteins are all involved in enzymatic reactions that sequentially add on malonyl-coenzyme-A molecules to the growing hydrocarbon chain during fatty acid biosynthesis (Heath et al., 2001). The AccABCD proteins form a multi-subunit enzyme responsible for the synthesis of malonyl-CoA from acetyl-CoA. Thus, all of these proteins are associated with fatty acid chain elongation, and they were overproduced, at the protein and/or transcript level, in the TIGR4 *yycG*::*kan*[pPL100] strain. On the other hand, both *fabH*, and *acpP*, showed a decrease in transcription in TIGR4 *yycG*::*kan*[pPL100] after the induction of *yycF.* The *acpP* gene encodes the acyl carrier protein (ACP), which is the fundamental starting point for fatty acid biosynthesis, and FabH is responsible for condensing acetyl-CoA with malonyl-ACP to form α-ketobutyryl-ACP, the starter molecule for subsequent chain elongation. Therefore, it appears that genes involved in fatty acid starter unit production have reduced expression after *yycF* induction in the absence of YycG, whereas those involved in chain elongation have elevated expression, as previously observed with TIGR4[pPL100] (Mohedano et al., 2005).

As mentioned above, induction of *yycF* in TIGR4 *yycG*::*kan*[pPL100] resulted in a reduction of transcription of FabT (**Figure 2A**). FabT belongs to the MarR family of transcriptional regulators (Alekshun et al., 2001), and directly controls the expression of the two *fabTH-acpP* and *fabKDGF-accB-fabZ-accCDA* operons by repression of their transcription (Lu and Rock, 2006). In fact, overexpression of FabT in *S. pneumoniae* results in an increase of the C18:C16 ratio of unsaturated and saturated fatty acids (Lu and Rock, 2006), the same phenotype that we previously observed in TIGR4 upon overexpression of YycF (Mohedano et al., 2005). Our results demonstrate that NP-YycF is able to bind specifically to the promoter region of *fabTH-acpP* operon and to inhibit *in vitro* transcription driven from this promoter in a dose dependent manner. These results support the phenotypes previously observed (Mohedano et al., 2005; Lu and Rock, 2006) and indicate that the non-phosphorylated YycF specifically represses the expression of FabT regulator by direct binding to its promoter region. In fact, there is a sequence hexamer (5′-TGTAAA-3′) located between the P_FabT_ and the ATG translation start codon of *fabT* (**Figure 4**), similar to the consensus sequence previously described for binding of pneumococcal YycF (Ng et al., 2005). This sequence could be the binding site for a monomeric NP-YycF. Its binding to this region would impair the elongation of the *fabT* mRNA by the RNA polymerase thus repressing the FabT expression. Therefore, the overall results indicate that NP-YycF, rather than P-YycF, regulates fatty acid synthesis by controlling expression of FabT.

The band-shift experiments revealed that *in vitro* a three thousand fold excess of NP-YycF is required for interaction with the promoter region of the *fabTH-acpP* operon (**Figure 4A**). This may well be an overestimation due to the high salt concentration used in the assay which is necessary for YycF solubility, which might decrease the affinity of the regulator for the DNA. Determination of the number of chromosomally encoded YycF molecules in the control strain TIGR4[pLS1RGFP] revealed that there are approximately 565 molecules of YycF per bacterial cell (Supplementary Figure 3), which, according to the band-shift experiments, would not be sufficient to affect the expression of the *fab* operons. However, transient upregulation and/or dephosphorylation of YycF may occur under certain physiological conditions that could temporarily increase levels of NP-YycF thus allowing binding to *fabT* promoter. The regulation by NP-YycF would need to be transient, because an accumulation of free fatty acids would be deleterious for the cells. Our inability to subject *S. pneumoniae* to continuous induction of YycF in the absence of YycG supports this hypothesis. The ability of YycF to control cell wall metabolism even in the absence of YycG would explain why, in streptococci, the response regulator, but not the histidine kinase, is essential for cell survival. However, it has been shown that YycG exhibits a strong phosphatase activity that mediates dephosphorylation of P-YycF in biochemical reactions (Gutu et al., 2010; Huynh and Stewart, 2011) and that this activity is strongly active in exponentially growing cells, limiting cross-talk (Wayne et al., 2012). Consequently, a balance between P-YycF and NP-YycF accomplished by the YycG, is likely to occur in pneumococci, in which each form of YycF may regulate different pathways.

Current knowledge supports that P-YycF in *S. pneumoniae* (Ng et al., 2003, 2005; Mohedano et al., 2005) as in other Gram-positive bacteria, regulates expression of genes involved in cell wall metabolism and cell division. However, differences in regulation of these processes seem to occur in different *S. pneumoniae* serotypes. We have detected (Mohedano et al., 2005 and unpublished results) that in TIGR4 (serotype 4) TCS induction causes a decrease of *mreDC* transcription, whereas no effect on expression of this operon was observed upon YycFG depletion in the R6 strain (uncapsulated derivative of D39 seroptype 2; Ng et al., 2003, 2005). This is an operon that encodes proteins involved in cell morphogenesis in *E. coli* (Osborn and Rothfield, 2007) and *B. subtilis* (Hayhurst et al., 2008). However, little is known of the role of *mreCD* in *S. pneumoniae.* It has been reported that in the D39 and R6 strains *mreCD* can be deleted, and perturbations of expression of the operon did not contribute to cell division defects of *pcsB* mutants (Barent et al., 2009). Furthermore, expression of the virulence factor PspA (pneumococcal protein surface A) was altered by YycFG depletion in R6 (Ng et al., 2003, 2005) though not affected by overexpression of the TCS in TIGR4 (Mohedano et al., 2005 and unpublished results). Finally, studies of TCS mutants in animal models of pneumonia caused attenuation of infection in some cases and had no effect in others, and it has been proposed that this could be due to differences among the pneumococcal strains or growth conditions (Paterson et al., 2006). These differences observed between pneumococcal strains regarding the role of TCS and other regulatory mechanisms may influence the virulence of different seroptypes, and could also account for the differences in pathogenicity.

## Conclusion

This work provides evidence that the YycFG TCS of *S. pneumoniae* controls fatty acid biosynthesis by repressing expression of the FatT transcriptional repressor. These results expand the role of YycFG in this bacterium, regulating not only the cell wall homeostasis but also membrane composition. Therefore the YycFG, as other TCS, has an important role in pneumococcus, in adaptation to environmental changes. In addition, it supports that the monomeric NP-YycF is also able to regulate gene expression adding a new dimension to this RR, which may act as a phosphorylated dimer or as a non-phosphorylated monomer regulating different pathways. This discovery may have important implications in the search for new antimicrobials targeting the YycFG system, in which previously all efforts were made toward inhibition of dimerization and/or phosphorylation.

## Author Contributions

MM contributed to the molecular cloning, band shift experiments and proteomic studies. AF performed the transcriptional gene expression analysis. MA purified and characterized the YycF protein and performed the *in vitro transcriptional analysis.* JW participate in study conception and corrected the manuscript. PL participated in study conception, data interpretation and wrote the manuscript. All authors have read and approved the final manuscript.

## Conflict of Interest Statement

The authors declare that the research was conducted in the absence of any commercial or financial relationships that could be construed as a potential conflict of interest.

## Footnotes

1 http://www.tigr.org

2 http://www.matrixscience.com/
