# Supplementary material for: The Response Regulator YycF Inhibits Expression of the Fatty Acid Biosynthesis Repressor FabT in Streptococcus pneumoniae
Source: Front Microbiol. 2016 Aug 25;7:1326. doi: 10.3389/fmicb.2016.01326 (PMC4996995; doi:10.3389/fmicb.2016.01326)
Supplement: Supplementary file 1 [file Table_1.DOCX]

Supplementary Material

**The response regulator** **YycF inhibits expression of the fatty acid biosynthesis repressor FabT in *Streptococcus pneumoniae***

Maria Luz Mohedano^1^, Mónica Amblar^2^, Alicia de la Fuente^1^, Jerry M. Wells^3^ and Paloma López^1*^

^1^Laboratorio de Biología Molecular de Bacterias Gram positivas, Departamento de [Microbiología Molecular y Biología de las Infecciones](http://www.cib.csic.es/es/departamento.php?iddepartamento=10), Centro de Investigaciones Biológicas, CSIC, Madrid, Spain.

^2^Unidad de Patología Molecular del Neumococo, Centro Nacional de Microbiología, Instituto de Salud Carlos III, Majadahonda, Madrid, Spain.

^3^Host–Microbe Interactomics, Animal Sciences Department, University of Wageningen, Wageningen, The Netherlands.

***Correspondence:** Dr. Paloma López. Centro de Investigaciones Biológicas. Ramiro de Maeztu 9, 28040 Madrid, Spain.

plg@cib.csic.es

**Supplementary Table S1.** The proteins affected by pPL100 and/or pPL101 overexpression detected by 2D gel analysis

| **Spots** | **10 min of induction** | | | **30 min of induction** | | | **Long-term induction** | |  |
| --- | --- | --- | --- | --- | --- | --- | --- | --- | --- |
|  | **TIGR4**  ***yycG*::*kan*[pPL100]** | **TIGR4[pPL101]** | **TIGR4[pLS1RGFP]** | **TIGR4**  ***yycG*::*kan*[pPL100]** | **TIGR4[pPL101]** | **TIGR4[pLS1RGFP]]** | **TIGR4[pPL101]** | **TIGR4[pLS1RGFP]** | **TIGR4 No** |
| YycF | 8210±90 | 9128±376 | <522 | 10295±844 | 12627±403 | <446 | 16368±111 | <408 | Sp1227 |
| YycF^*^ | 2589±183 | 2352±357 | <57 | 3399±414 | 3969±349 | <58 | 4412±947 | <59 | Sp1227 |
| FabK | 4378±517 | 2130±336 | 1970±202 | 4612±442 | 2069±287 | 1998±332 | 1798±182 | 1684±273 | Sp0419 |
| FabD | 2308±158 | 1310±18 | 1162±267 | 2745±80 | 1065±264 | 984±303 | 1232±269 | 1370±204 | Sp0420 |
| FabG | 7378±614 | 3303±583 | 2974±403 | 9340±1489 | 3652±113 | 3311±594 | 2893±392 | 2900±541 | Sp0421 |
| FabF^1^ | 7133±876 | 4091±1147 | 3733±907 | 7788±904 | 3314±980 | 3339±506 | 3500±1433 | 3450±191 | Sp0422 |
| FabF1^1^ | 1603±162 | 616±184 | 454±71 | 1206±349 | 461±158 | 434±112 | 230±106 | 339±54 | Sp0422 |
| FabF2^1^ | 836±102 | 547±110 | 512±154 | 1075±61 | 496±136 | 394±97 | 368±301 | 230±94 | Sp0422 |
| FabF3^1^ | 1125±114 | 566±155 | 531±143 | 970±278 | 529±377 | 404±70 | 339±245 | 255±54 | Sp0422 |
| AccB | 2158±28 | 1292±225 | 1169±19 | 2393±333 | 1205±242 | 1196±53 | 1283±102 | 1098±121 | Sp0423 |
| AccC | 1825±275 | 728±26 | 960±252 | 1695±113 | 762±143 | 662±140 | 770±65 | 652±68 | Sp0425 |
| AccA | 2217±396 | 971±0.75 | 748±61 | 2589±479 | 1060±398 | 1206±355 | 689±90 | 809±82 | Sp0427 |
| Kan | 5383±196 | <5 | <5 | 4353±424 | <5 | <5 | <5 | <5 | <5 |

AccA, Acetyl-CoA carboxylase carboxyl transferase A subunit; AccB, Acetyl-CoA carboxylase biotin carboxyl carrier protein; AccC, Acetyl-CoA carboxylase biotin carboxylase; FabD, Malonyl CoA-acyl carrier protein transacylase; FabF, Oxoacyl-(acyl-carrier-protein) synthase II; FabG, 3-oxoacyl-(acyl-carrier-protein) reductase; FabK, Enoyl-(acyl-carrier-protein) reductase.

^1^Different isoforms of FabF.
