# Supplementary material for: The Response Regulator YycF Inhibits Expression of the Fatty Acid Biosynthesis Repressor FabT in Streptococcus pneumoniae
Source: Front Microbiol. 2016 Aug 25;7:1326. doi: 10.3389/fmicb.2016.01326 (PMC4996995; doi:10.3389/fmicb.2016.01326)
Supplement: Supplementary file 2 [file Image_1.PDF]

Supplementary Material

The response regulator YycF inhibits expression of the fatty acid biosynthesis repressor FabT in *Streptococcus pneumoniae*

Maria Luz Mohedano[1], Mónica Amblar[2], Alicia de la Fuente[1], Jerry M. Wells[3] and Paloma López[1*]

[1]Laboratorio de Biología Molecular de Bacterias Gram positivas, Departamento de Microbiología Molecular y Biología de las Infecciones, Centro de Investigaciones Biológicas, CSIC, Madrid, Spain.

[2]Unidad de Patología Molecular del Neumococo, Centro Nacional de Microbiología, Instituto de Salud Carlos III, Majadahonda, Madrid, Spain.

[3]Host–Microbe Interactomics, Animal Sciences Department, University of Wageningen, Wageningen, The Netherlands.

***Correspondence:** Dr. Paloma López. Centro de Investigaciones Biológicas. Ramiro de Maeztu 9, 28040 Madrid, Spain.

plg@cib.csic.es

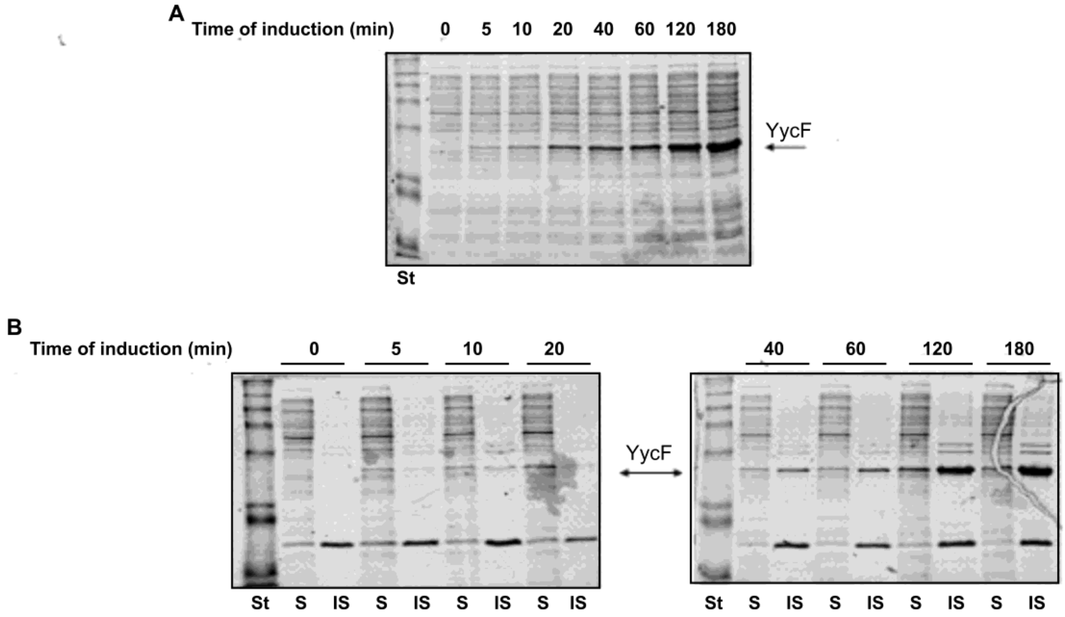

Supplementary Figure 1. Analysis of $(His)_6$-YycF production in *E. coli*.

Cultures of *E. coli* M15[pREP4][pPL102] were induced with IPTG at 30ºC for the times indicated in the figure. Total protein extracts were prepared from 1 ml of the induced cultures by sedimenting the cells and resuspending in 100 μl of a buffer containing 50 mM Tris-HCl pH 6.8, 2% SDS, 2 mM de EDTA and 143 mM β-mercaptoetanol and heating at 100 ºC for 10 min. For analysis of $(His)_6$-YycF solubility, 1 ml of induced cultures were sedimented and resuspended in 100 μl of a buffer containing 50 mM Tris-HCl pH 7.5, 2 mM EDTA and lysozyme at 100 μg ml^{-1}. Then, after one cycle of freezing in a mixture of dry ice and ethanol and thawing at 15ºC, cells were lysed by incubation at 30ºC for 10 min. Then, the cells extracts were passed through a needle (25G 5/8 0.5 x 16 mm) to decrease the viscosity and the soluble and insoluble protein fractions were fractionated by centrifugation at 12,000 x g during 10 min. The proteins were analysed by 15% SDS-PAGE, and the gels were stained with Coomassie Blue. (A) Total protein extracts from IPTG-induced cultures of *E. coli*. (B) Analysis of soluble (S) and insoluble (IS) form of the recombinant protein at different times of induction. St, molecular weight standard.
