# Supplementary material for: The Response Regulator YycF Inhibits Expression of the Fatty Acid Biosynthesis Repressor FabT in Streptococcus pneumoniae
Source: Front Microbiol. 2016 Aug 25;7:1326. doi: 10.3389/fmicb.2016.01326 (PMC4996995; doi:10.3389/fmicb.2016.01326)
Supplement: Supplementary file 3 [file Image_2.PDF]

Supplementary Material

The response regulator YycF inhibits expression of the fatty acid biosynthesis repressor FabT in *Streptococcus pneumoniae*

Maria Luz Mohedano[1], Mónica Amblar[2], Alicia de la Fuente[1], Jerry M. Wells[3] and Paloma López[1*]

[1]Laboratorio de Biología Molecular de Bacterias Gram positivas, Departamento de Microbiología Molecular y Biología de las Infecciones, Centro de Investigaciones Biológicas, CSIC, Madrid, Spain.

[2]Unidad de Patología Molecular del Neumococo, Centro Nacional de Microbiología, Instituto de Salud Carlos III, Majadahonda, Madrid, Spain.

[3]Host–Microbe Interactomics, Animal Sciences Department, University of Wageningen, Wageningen, The Netherlands.

***Correspondence:** Dr. Paloma López. Centro de Investigaciones Biológicas. Ramiro de Maeztu 9, 28040 Madrid, Spain.

plg@cib.csic.es

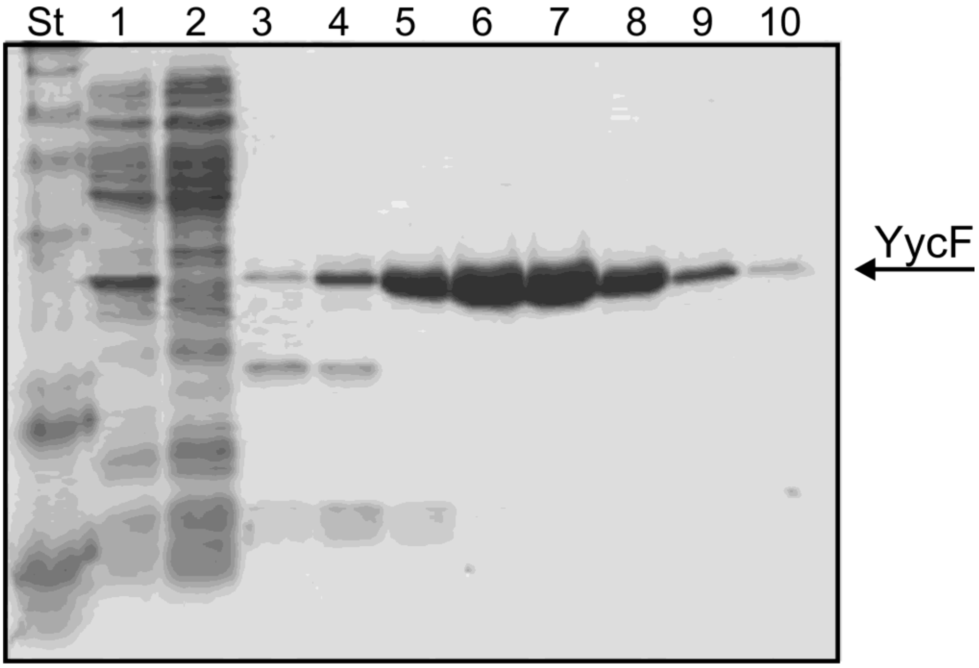

Supplementary Figure 2. Purification of $(His)_6$-YycF.

Proteins were analyzed by 15% SDS-PAA. Lanes: St, molecular weight standard, 1, total extract loaded in a HisTrap HP column; 2, proteins eluting during washing with 20 mM imidazole and 3-10, fractions containing the purified YycF eluted with 245 to 318 mM imidazole.
