# Supplementary material for: The Response Regulator YycF Inhibits Expression of the Fatty Acid Biosynthesis Repressor FabT in Streptococcus pneumoniae
Source: Front Microbiol. 2016 Aug 25;7:1326. doi: 10.3389/fmicb.2016.01326 (PMC4996995; doi:10.3389/fmicb.2016.01326)
Supplement: Supplementary file 4 [file Image_3.PDF]

Supplementary Material

The response regulator YycF inhibits expression of the fatty acid biosynthesis repressor FabT in *Streptococcus pneumoniae*

Maria Luz Mohedano[1], Mónica Amblar[2], Alicia de la Fuente[1], Jerry M. Wells[3] and Paloma López[1*]

[1]Laboratorio de Biología Molecular de Bacterias Gram positivas, Departamento de Microbiología Molecular y Biología de las Infecciones, Centro de Investigaciones Biológicas, CSIC, Madrid, Spain.

[2]Unidad de Patología Molecular del Neumococo, Centro Nacional de Microbiología, Instituto de Salud Carlos III, Majadahonda, Madrid, Spain.

[3]Host–Microbe Interactomics, Animal Sciences Department, University of Wageningen, Wageningen, The Netherlands.

*__Correspondence:__ Dr. Paloma López. Centro de Investigaciones Biológicas. Ramiro de Maeztu 9, 28040 Madrid, Spain.

plg@cib.csic.es

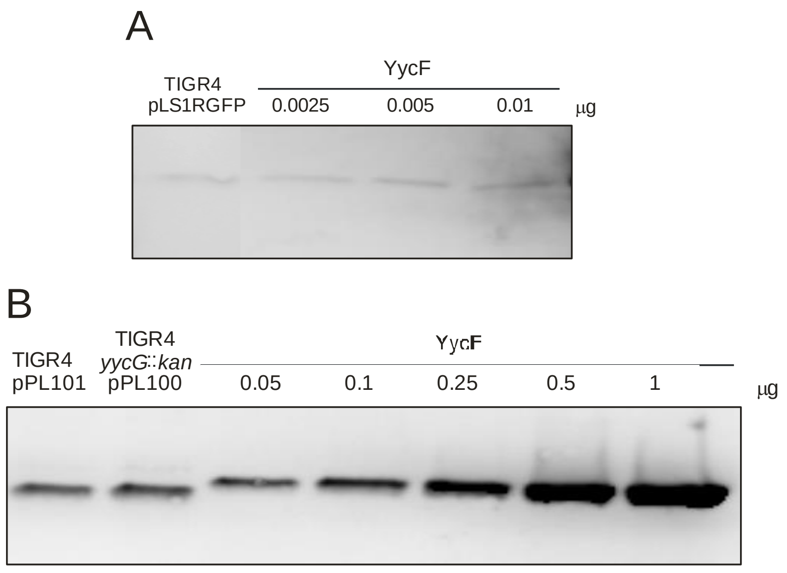

Supplementary Figure 3. Detection of YycF levels.

Total protein extracts of 10 min induced cultures of the indicated strain and purified $(His)_6$-YycF were fractionated by 15% SDS-PAA. The RR was detected by western blot hybridization. See details in Materials and Methods.
